# The predictive value comparison of the different nutritional assessment tools for postoperative delirium in elderly patients after non-cardiac surgery

**DOI:** 10.3389/fnut.2025.1673973

**Published:** 2025-10-21

**Authors:** Xuzhou Dang, Yefang Yang, Yu Liang, Yuting Liu, Wenjie Zhang, Xuesen Su

**Affiliations:** ^1^The College of Anesthesia, Shanxi Medical University, Taiyuan, Shanxi, China; ^2^Department of Anesthesiology, Datong Third People’s Hospital, Datong, Shanxi, China; ^3^Department of Anesthesiology, First Hospital of Shanxi Medical University, Taiyuan, Shanxi, China; ^4^The Anesthesiology Department of Shanxi Provincial People’s Hospital, Shanxi Medical University, Taiyuan, Shanxi, China

**Keywords:** nutritional assessment, elderly, non-cardiac surgery, postoperative delirium, predictive model, Mini nutritional assessment (MNA), C-reactive protein (CRP)

## Abstract

**Objective:**

This study aims to evaluate and compare the predictive performance of various nutritional assessment tools.

**Methods:**

This prospective observational study enrolled 315 elderly patients (≥65 years) scheduled for non-cardiac surgery at Shanxi Medical University First Hospital between March and May 2025. Preoperative data collected included demographics, laboratory indices, Geriatric Nutritional Risk Index (GNRI), Prognostic Nutritional Index (PNI), and Mini Nutritional Assessment (MNA). Postoperative delirium (POD) was diagnosed daily during the 7 days postoperatively using the 3-Minute Diagnostic Confusion Assessment Method (3D-CAM). Patients were stratified into Delirium (*n* = 54) and non-delirium (*n* = 249) groups. Logistic regression identified independent POD predictors. Subsequently, Receiver Operating Characteristic (ROC) curve analysis assessed predictive performance (AUC, sensitivity, specificity) of individual tools and combined models.

**Results:**

MNA and PNI scores were significantly lower in the delirium group compared to the non-delirium group (*p* < 0.05), while GNRI scores showed no significant difference. Multivariate analysis identified older age (OR = 1.07, 95% CI: 1.02–1.12), elevated CRP (OR = 1.06, 95% CI: 1.03–1.10), and lower MNA score (OR = 0.79, 95% CI: 0.70–0.88) as independent predictors of POD. ROC analysis revealed the continuous variable of MNA score as the superior single predictor (AUC = 0.741, 95% CI: 0.67–0.81), significantly outperforming PNI (AUC = 0.603, *p* = 0.008) and GNRI (AUC = 0.442, *p* < 0.001). The combined model including age, C-reactive protein (CRP), and MNA achieved the highest predictive accuracy (AUC = 0.810, 95% CI: 0.75–0.87; sensitivity 71%, specificity 80%), significantly better than other combinations. Adding PNI or GNRI did not further improve model performance.

**Conclusion:**

MNA is the most effective standalone nutritional tool for predicting POD in elderly non-cardiac surgery patients. A combined model incorporating age, CRP, and MNA score (AUC = 0.810) shows higher accuracy and improved clinical usefulness for preoperative risk stratification. This allows targeted interventions for high-risk individuals.

## Background

Rapid population aging has greatly increased the demand for surgeries in the elderly. Therefore, preventing postoperative complications has become a critical clinical priority. Postoperative delirium (POD) involves acute neurocognitive dysfunction (1, 2) and affects 13–50% of elderly patients undergoing non-cardiac surgery (3, 4). POD is strongly associated with prolonged hospitalization, increased healthcare costs, long-term cognitive impairment, and elevated mortality (5–7). Current treatment options remain limited and often ineffective (8, 9), highlighting the importance of early prevention and preoperative identification of high-risk patients. Emerging evidence suggests that preoperative malnutrition is an independent risk factor for POD (10–13), with a reported prevalence of 30–60% in hospitalized elderly populations (14, 15). Commonly used nutritional assessment tools include the Mini Nutritional Assessment (MNA), Prognostic Nutritional Index (PNI), and Geriatric Nutritional Risk Index (GNRI) (16, 17). MNA, developed by Cohendy R et al. in the 1990s (18), is a multidimensional tool encompassing anthropometric measurements, global assessment, a dietary questionnaire, and a subjective assessment (maximum score 30). PNI, calculated from serum albumin and lymphocyte count, is widely used to predict surgical risk, postoperative complications, and prognosis (19, 20). GNRI, derived from serum albumin and the ratio of actual to ideal body weight (21), assesses nutritional risk. These nutritional assessment tools play a crucial role in assessing preoperative nutritional status and predicting postoperative delirium (22–26). Although nutritional assessment tools have shown some effectiveness in evaluating postoperative delirium, several issues remain. For instance, assessment results may vary among different tools, and the predictive value for delirium in specific populations and after specific surgeries requires further validation. This prospective cohort study addresses two key questions: (1) To quantitatively compare the predictive performance of MNA, PNI, and GNRI for POD in elderly non-cardiac surgery patients; (2) To determine whether a combined model significantly outperforms individual tools. This study aims to develop a practical risk stratification strategy that improves perioperative resource allocation and patient outcomes.

## Methods

### Study design and population

This single-center prospective observational study (Supplementary Table 1) was conducted at Shanxi Medical University First Hospital from March to May of 2025. Inclusion criteria: age ≥65 years; elective non-cardiac surgeries (orthopedics, general surgery, urology, thoracic surgery); ASA classification I–III; patients conscious preoperatively and able to cooperate with nutritional assessment and other tests; estimated hospital stay over 3 days. Exclusion criteria included: pre-existing delirium, Alzheimer’s disease, or severe psychiatric disorders; severe hearing or language impairment hindering communication; admission to the ICU after the operation; life expectancy under 6 months (e.g., terminal cancer, end-stage liver or renal failure); major intraoperative complications (e.g., massive hemorrhage, cardiac arrest) or death within 24 h postoperatively; and patient or family refusal to participate.

### Ethical approval and registration

The study was approved by the Institutional Review Board of The First Hospital of Shanxi Medical University (Approval Number KYLL-2025-103), and was conducted in accordance with the principles of the Declaration of Helsinki. The trial was registered with the Chinese Clinical Trial Registry (ChiCTR2500104264). Written informed consent was obtained from all participants.

### Data collection

Preoperative assessment was conducted within 48 h of admission. Trained research nurses collected demographics, including age and sex, American Society of Anesthesiologists Physical Status Classification (ASA PS), body mass index (BMI), education level, Charlson Comorbidity Index (CCI), and living status. “Living alone” was defined as residing without family or caregivers. Trained research nurses screened for preoperative delirium using 3-Minute Diagnostic Confusion Assessment Method (3D-CAM). Laboratory data, including hemoglobin (Hb), lymphocyte count (LYM), serum albumin (Alb), creatinine (Cr), and C-reactive protein (CRP), were extracted from electronic medical records within 24 h before surgery. Surgical data included anesthesia type (general or neuraxial), anesthesia duration, and surgical duration, all measured in minutes; data accuracy was verified by dual entry.

### Nutritional assessment

MNA, PNI, and GNRI were selected for this study. MNA evaluated several factors, including dietary intake, weight loss, mobility, psychological state, and anthropometric measurements such as BMI and calf circumference. The scores were categorized as follows: ≥24 indicates well-nourished; 17–23.5 indicates risk of malnutrition; and <17 indicates malnourished (18). PNI was calculated using the formula: PNI = Alb (g/dL) + 5 × LYM (10^9^/L), where Alb is serum albumin concentration and LYM is lymphocyte count. Categories were defined as follows: >38 (normal); 35–38 (moderate risk); and <35 (severe risk) (20). GNRI was calculated as GNRI = [1.489 × Alb (g/L)] + [41.7 × (Actual Weight ÷ Ideal Weight)], where Alb is expressed in g/L. The ideal weight was calculated using the Lorentz formula: for males, Ideal Weight = 0.75 × height (cm) − 62.5; for females, Ideal Weight = 0.60 × height (cm) − 40. GNRI categories included >98 (no risk); 92–98 (mild risk); 82 to less than 92 (moderate risk); and <82 (severe risk) (21).

### POD assessment

POD was assessed twice daily (morning and afternoon) from postoperative day 1 through 7 using the 3D-CAM by a trained anesthesiologist (27). POD diagnosis required the presence of Feature 1: acute change/fluctuating course, Feature 2: inattention, and either Feature 3: altered level of consciousness or Feature 4: disorganized thinking.

### Statistical analysis and sample size

Data were analyzed using SPSS 26.0 and R software, with normality assessed by the Shapiro–Wilk test. Normally distributed continuous data are presented as mean ± standard deviation (SD) and compared using Student’s *t*-test. Non-normally distributed data are presented as median (Q1–Q3) and compared using the Mann–Whitney *U* test. Categorical data were presented as *n* (%) and compared using Chi-square or Fisher’s exact test. Univariate logistic regression identified variables associated with POD (*p* < 0.05), which were then entered into the multivariate logistic regression to identify independent predictors. ROC curve analysis determined the area under the curve (AUC), sensitivity, specificity, and optimal cut-off value, based on the Youden index, for individual tools and combined models. DeLong’s (28) test compared AUC differences, with *p* < 0.05 considered significant. To further examine dose–response relationship between scores of nutritional assessment and the risk of POD, a restricted cubic spline regression (RCS) model with three knots (5th, 50th, and 95th percentiles) was employed. Tests for nonlinearity were performed using the likelihood ratio test. In addition, subgroup analyses stratified by gender, age, ASA PS, and living alone or not were conducted to explore the robustness of the research results and the influencing factors.

Based on an expected POD incidence of 20% (3, 4), a target AUC ≥ 0.80, *α* = 0.05, and *β* = 0.10, the required sample size was calculated as 267 (requiring ≥54 events) using the R pmsampsize package. Accounting for a 15% attrition rate, 315 patients were recruited.

## Results

### Characteristics of participants

**Figure 1:**
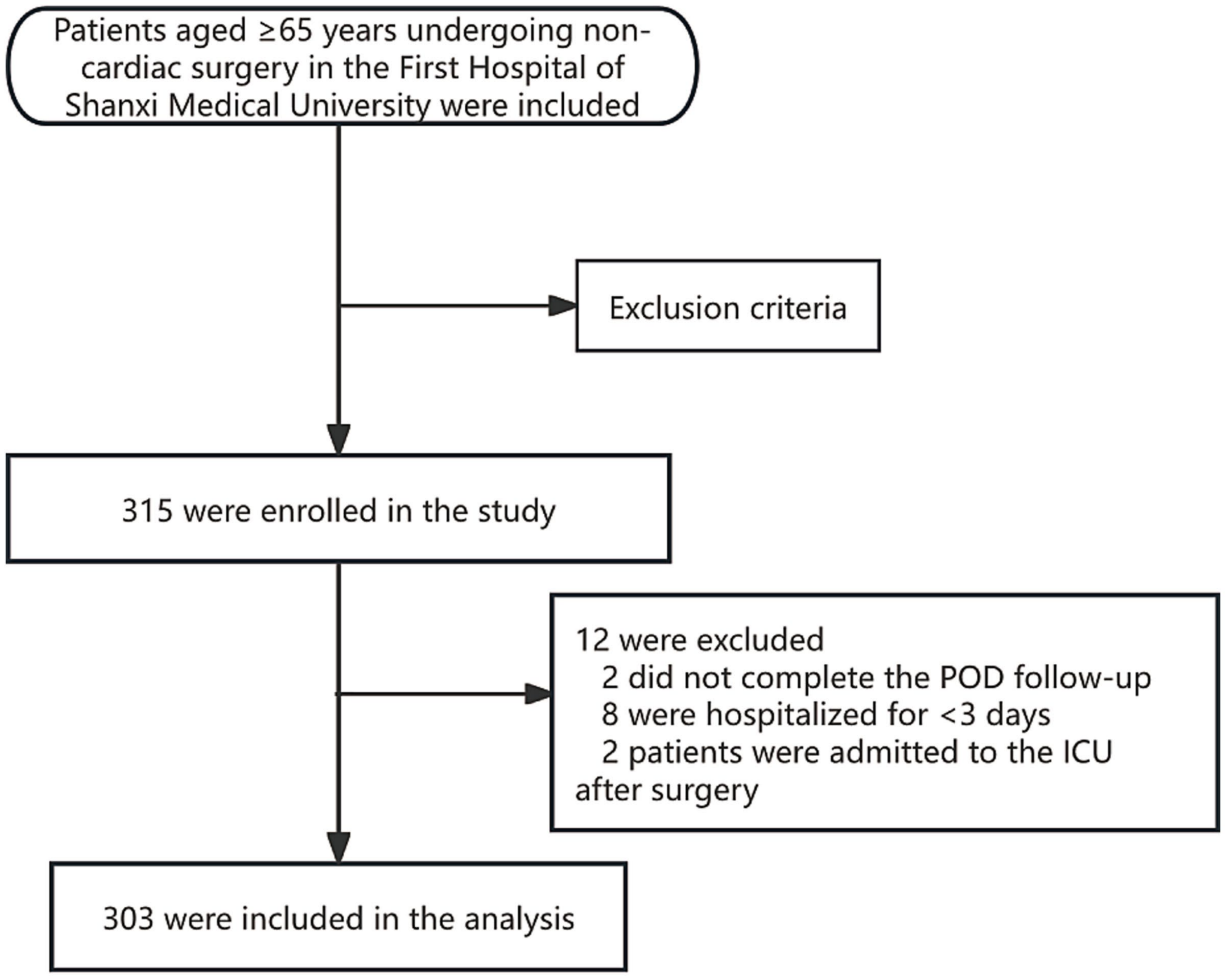
Flow chart of case selection in this study.

A total of 315 patients were enrolled. Twelve were excluded (Figure 1), leaving 303 patients for analysis. The incidence of POD was 17.82% (*n* = 54). Table 1 compares baseline characteristics between the Delirium and Non-Delirium groups. No significant differences were found between the delirium and non-delirium groups in years of education (*Z* = −0.12, *p* = 0.904), CCI (*Z* = −0.19, *p* = 0.853), anesthesia time (*Z* = −1.93, *p* = 0.054), gender (*χ*^2^ = 2.44, *p* = 0.118), anesthesia method (*χ*^2^ = 2.23, *p* = 0.136), hearing loss (*χ*^2^ = 1.75, *p* = 0.186), or visual impairment (*χ*^2^ = 3.12, *p* = 0.078). However, compared with the non-delirium group, patients in the delirium group had a lower BMI (*t* = 2.87, *p* = 0.004), were older (*Z* = 4.51, *p* < 0.001), had longer operative times (*Z* = 2.02, *p* = 0.043), higher ASA PS (*χ*^2^ = 9.06, *p* = 0.003), and a higher proportion living alone (*χ*^2^ = 25.91, *p* < 0.001). Preoperative blood tests showed that white blood cell count (*Z* = −3.80, *p* < 0.001), creatinine (*Z* = −3.13, *p* = 0.002), and CRP (*Z* = −5.90, *p* < 0.001) were significantly higher in patients with postoperative delirium. No significant differences were observed in red blood cell count (*Z* = −0.60, *p* = 0.545), hemoglobin (*Z* = −0.25, *p* = 0.806), or albumin (*Z* = −1.23, *p* = 0.217). Regarding nutrition-related indicators, the delirium group had significantly lower MNA (*Z* = −5.59, *p* < 0.001) and PNI scores (*Z* = −2.38, *p* = 0.018) compared to the non-delirium group, while GNRI scores did not differ significantly (*Z* = −1.34, *p* = 0.181).

**Table 1:** Baseline characteristics of the study population.

Variables	Total (*n* = 303)	No-POD (*n* = 249)	POD (*n* = 54)	Z/t/*χ*^2^ value	*p* value
BMI, (kg/m^2^)	24.24 ± 3.36	24.50 ± 3.28	23.07 ± 3.49	*t* = 2.87	0.004
Age, (year)	70.00 (65.00, 75.00)	69.00 (65.00, 74.00)	76.50 (68.00, 81.00)	*Z* = −4.51	<0.001
Education years	6.00 (4.00, 8.00)	6.00 (4.00, 8.00)	6.00 (4.25, 8.00)	*Z* = −0.12	0.904
MNA score	24.00 (22.00, 25.00)	24.00 (22.00, 25.00)	22.00 (20.00, 23.00)	*Z* = −5.59	<0.001
PNI score	45.95 (42.78, 48.88)	46.05 (43.80, 48.85)	43.90 (37.60, 48.80)	*Z* = −2.38	0.018
GNRI score	106.82 (98.32, 115.48)	106.32 (98.73, 114.02)	108.76 (96.66, 139.78)	*Z* = −1.34	0.181
CCI	0.00 (0.00, 1.00)	0.00 (0.00, 1.00)	0.00 (0.00, 1.75)	*Z* = −0.19	0.853
Anesthesia time (min)	119.50 (100.00, 156.50)	118.00 (100.00, 144.00)	121.50 (101.75, 200.00)	*Z* = −1.93	0.054
Operation time (min)	91.00 (72.00, 125.00)	90.00 (71.50, 122.00)	95.00 (77.00, 178.75)	*Z* = −2.02	0.043
WBC (×10^9^/L)	6.00 (4.90, 7.60)	5.80 (4.90, 7.40)	7.05 (5.80, 8.45)	*Z* = −3.80	<0.001
RBC (×10^9^/L)	4.25 (3.84, 4.54)	4.25 (3.89, 4.52)	4.30 (3.69, 4.58)	*Z* = −0.60	0.545
Hb (g/L)	130.00 (117.00, 139.00)	130.00 (117.00, 138.00)	131.50 (117.00, 139.75)	*Z* = −0.25	0.806
Cr (μmol/L)	65.80 (51.10, 76.90)	62.50 (51.00, 74.00)	72.30 (61.90, 86.97)	*Z* = −3.13	0.002
CRP (mg/L)	2.80 (1.54, 7.65)	2.36 (1.45, 5.57)	8.89 (3.82, 28.04)	Z = −5.90	<0.001
Alb (g/L)	38.60 (35.90, 40.70)	38.60 (36.60, 40.70)	38.50 (32.55, 41.20)	*Z* = −1.23	0.217
Gender, *n* (%)				*χ*^2^ = 2.44	0.118
Female	147 (48.51)	126 (50.60)	21 (38.89)		
Male	156 (51.49)	123 (49.40)	33 (61.11)		
ASA PS, *n* (%)				*χ*^2^ = 9.06	0.003
II	146 (48.18)	130 (52.21)	16 (29.63)		
III	157 (51.82)	119 (47.79)	38 (70.37)		
Anesthesia method, *n* (%)				*χ*^2^ = 2.23	0.136
General anesthesia	281 (92.74)	234 (93.98)	47 (87.04)		
Intraspinal anesthesia	22 (7.26)	15 (6.02)	7 (12.96)		
Alone, *n* (%)				*χ*^2^ = 25.91	<0.001
No	228 (75.25)	202 (81.12)	26 (48.15)		
Yes	75 (24.75)	47 (18.88)	28 (51.85)		
Hearing Impairment, *n* (%)				*χ*^2^ = 1.75	0.186
No	59 (19.47)	45 (18.07)	14 (25.93)		
Yes	244 (80.53)	204 (81.93)	40 (74.07)		
Impaired vision, *n* (%)				*χ*^2^ = 3.12	0.078
No	63 (20.79)	47 (18.88)	16 (29.63)		
Yes	240 (79.21)	202 (81.12)	38 (70.37)		

BMI, body mass index; MNA, Mini Nutritional Assessment; PNI, Prognostic Nutritional Index; GNRI, Geriatric Nutrition Risk Index; CCI, Charlson Comorbidity Index; WBC, White Blood Cell; RBC, Red Blood Cell; Hb, hemoglobin; Cr, creatinine; CRP, C-reactive protein; Alb, Albumin; ASA PS, American Society of Anesthesiologists Physical Status Classification; POD: Postoperative Delirium.

### Univariate and multivariate analysis of POD predictors

Univariate Logistic Regression results are shown in Table 2. Significant predictors of POD (*p* < 0.05) included older age (OR = 1.11, 95% CI: 1.06–1.15), lower BMI (OR = 0.88, 95% CI: 0.80–0.96), lower MNA score (OR = 0.78, 95% CI: 0.71–0.86), lower PNI (OR = 0.93, 95% CI: 0.88–0.98), higher GNRI (OR = 1.02, 95% CI: 1.01–1.03), longer surgical duration (OR = 1.01, 95% CI: 1.01–1.01), higher CRP (OR = 1.08, 95% CI: 1.05–1.11), lower albumin (OR = 0.93, 95% CI: 0.87–0.99), living alone (OR = 4.63, 95% CI: 2.49–8.61), and ASA III versus II (OR = 2.59, 95% CI: 1.38–4.90). Multivariate Logistic Regression results are presented in Table 3. The independent predictors retained in the final model were older age (OR = 1.07, 95% CI: 1.02–1.12), higher CRP (OR = 1.06, 95% CI: 1.03–1.10), and lower MNA score (OR = 0.79, 95% CI: 0.70–0.88).

**Table 2:** Univariate logistic regression results.

Variables	*β*	S.E.	*Z*	*p*	OR (95% CI)
Age	0.10	0.02	4.74	<0.001	1.11 (1.06 ~ 1.15)
BMI	−0.13	0.05	−2.80	0.005	0.88 (0.80 ~ 0.96)
Education years	−0.01	0.05	−0.24	0.807	0.99 (0.90 ~ 1.09)
MNA score	−0.25	0.05	−4.86	<0.001	0.78 (0.71 ~ 0.86)
PNI score	−0.08	0.03	−2.73	0.006	0.93 (0.88 ~ 0.98)
GNRI score	0.02	0.01	3.44	<0.001	1.02 (1.01 ~ 1.03)
CCI	0.07	0.11	0.68	0.497	1.08 (0.87 ~ 1.34)
Anesthesia time	0.01	0.00	3.33	<0.001	1.01 (1.01 ~ 1.01)
Operation time	0.01	0.00	3.44	<0.001	1.01 (1.01 ~ 1.01)
WBC	0.16	0.05	3.05	0.002	1.18 (1.06 ~ 1.30)
RBC	−0.16	0.26	−0.60	0.546	0.85 (0.51 ~ 1.43)
Hb	0.00	0.01	0.35	0.727	1.00 (0.99 ~ 1.02)
Cr	0.01	0.00	1.65	0.098	1.01 (1.00 ~ 1.02)
CRP	0.07	0.01	5.38	<0.001	1.08 (1.05 ~ 1.11)
Alb	−0.07	0.03	−2.16	0.031	0.93 (0.87 ~ 0.99)
ASA PS					
II					1.00 (reference)
III	0.95	0.32	2.94	0.003	2.59 (1.38 ~ 4.90)
Anesthesia method					
General anesthesia					1.00 (reference)
Intraspinal anesthesia	0.84	0.48	1.74	0.082	2.32 (0.90 ~ 6.01)
Gender					
Female					1.00 (reference)
Male	0.48	0.31	1.55	0.120	1.61 (0.88 ~ 2.94)
Alone					
No					1.00 (reference)
Yes	1.53	0.32	4.84	<0.001	4.63 (2.49 ~ 8.61)
Hearing impairment					
No					1.00 (reference)
Yes	−0.46	0.35	−1.31	0.189	0.63 (0.32 ~ 1.26)
Impaired vision					
No					1.00 (reference)
Yes	−0.59	0.34	−1.75	0.080	0.55 (0.28 ~ 1.07)

BMI, body mass index; MNA, Mini Nutritional Assessment; PNI, Prognostic Nutritional Index; GNRI, Geriatric Nutrition Risk Index; CCI, Charlson Comorbidity Index; WBC, White Blood Cell; RBC, Red Blood Cell; Hb, hemoglobin; Cr, creatinine; CRP, C-reactive protein; Alb, Albumin; ASA PS, American Society of Anesthesiologists Physical Status Classification.

**Table 3:** Multivariate logistic regression results.

Variables	*β*	S.E.	*Z*	*p*	OR (95%CI)
Age	0.06	0.02	2.67	0.008	1.07 (1.02 ~ 1.12)
BMI	0.01	0.06	0.13	0.897	1.01 (0.89 ~ 1.14)
MNA score	−0.24	0.06	−4.16	<0.001	0.79 (0.70 ~ 0.88)
PNI score	−0.01	0.06	−0.08	0.933	0.99 (0.88 ~ 1.12)
GNRI score	−0.01	0.01	−0.79	0.431	0.99 (0.97 ~ 1.01)
Anesthesia time	−0.02	0.02	−0.98	0.327	0.98 (0.94 ~ 1.02)
Operation time	0.03	0.02	1.22	0.224	1.03 (0.98 ~ 1.07)
WBC	0.11	0.06	1.78	0.075	1.12 (0.99 ~ 1.27)
Alb	0.00	0.07	0.01	0.991	1.00 (0.87 ~ 1.15)
CRP	0.06	0.02	4.03	<0.001	1.06 (1.03 ~ 1.10)
ASA PS					
II					1.00 (reference)
III	0.33	0.43	0.76	0.449	1.39 (0.60 ~ 3.23)
Alone					
No					1.00 (reference)
Yes	0.67	0.41	1.66	0.097	1.96 (0.89 ~ 4.35)

BMI, body mass index; MNA, Mini Nutritional Assessment; PNI, Prognostic Nutritional Index; GNRI, Geriatric Nutrition Risk Index; WBC, White Blood Cell; CRP, C-reactive protein; Alb, Albumin; ASA PS, American Society of Anesthesiologists Physical Status Classification.

**Figure 2:**
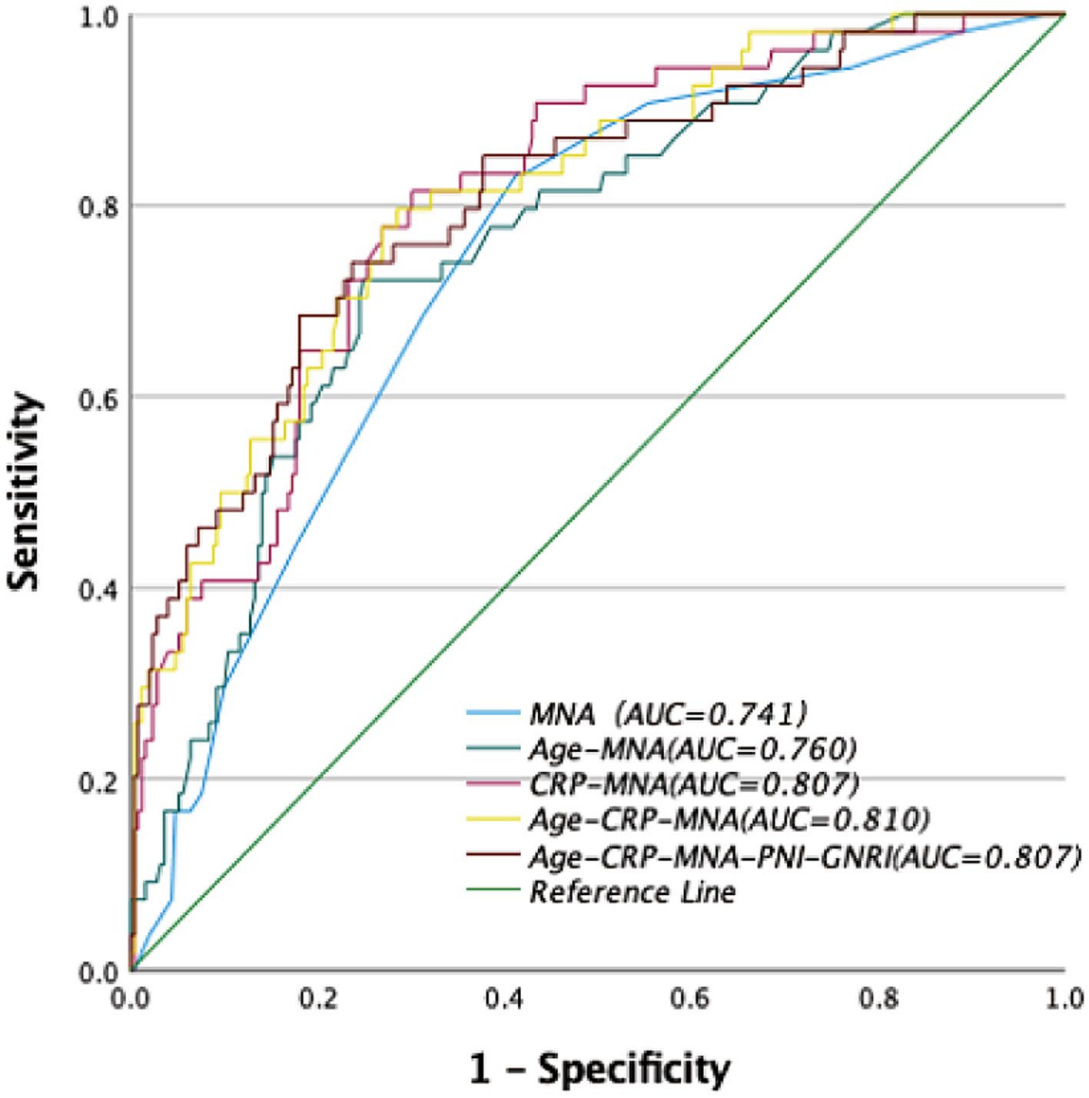
Receiver operator curve (ROC) of nutritional assessments scores for the risk of postoperative delirium (POD). MNA, Mini Nutritional Assessment; PNI, Prognostic Nutritional Index; GNRI, Geriatric Nutritional Risk Index.

The results of further RCS analysis showed the significant non-linear relationships between the scores of MNA, PNI, and GNRI and the risk of POD (Figures 2A–C).

### Predictive performance of nutritional tools and models

MNA demonstrated the highest predictive accuracy with an AUC of 0.741 (95% CI: 0.67–0.81), sensitivity of 41%, and specificity of 17% at the cutoff of 23.25. The optimal cut-off value for MNA (≤24) was determined by maximizing the Youden index in our cohort, consistent with its established classification for malnutrition risk. This performance was significantly superior to PNI (AUC = 0.603, 95% CI: 0.51–0.70; *p* = 0.008) and GNRI (AUC = 0.442, 95%CI:0.34–0.54; *p* < 0.001).

Among two-factor models, the combination of CRP and MNA yielded the highest AUC of 0.807 (95% CI: 0.75–0.87), with sensitivity of 70% and specificity of 81%.

Three-Factor Model (“Age + CRP + MNA”): This model achieved optimal predictive performance, with an AUC of 0.810 (95% CI: 0.75–0.87), sensitivity of 71%, and specificity of 80%. It was significantly better than any single tool or any other combination tested.

**Figure 3:**
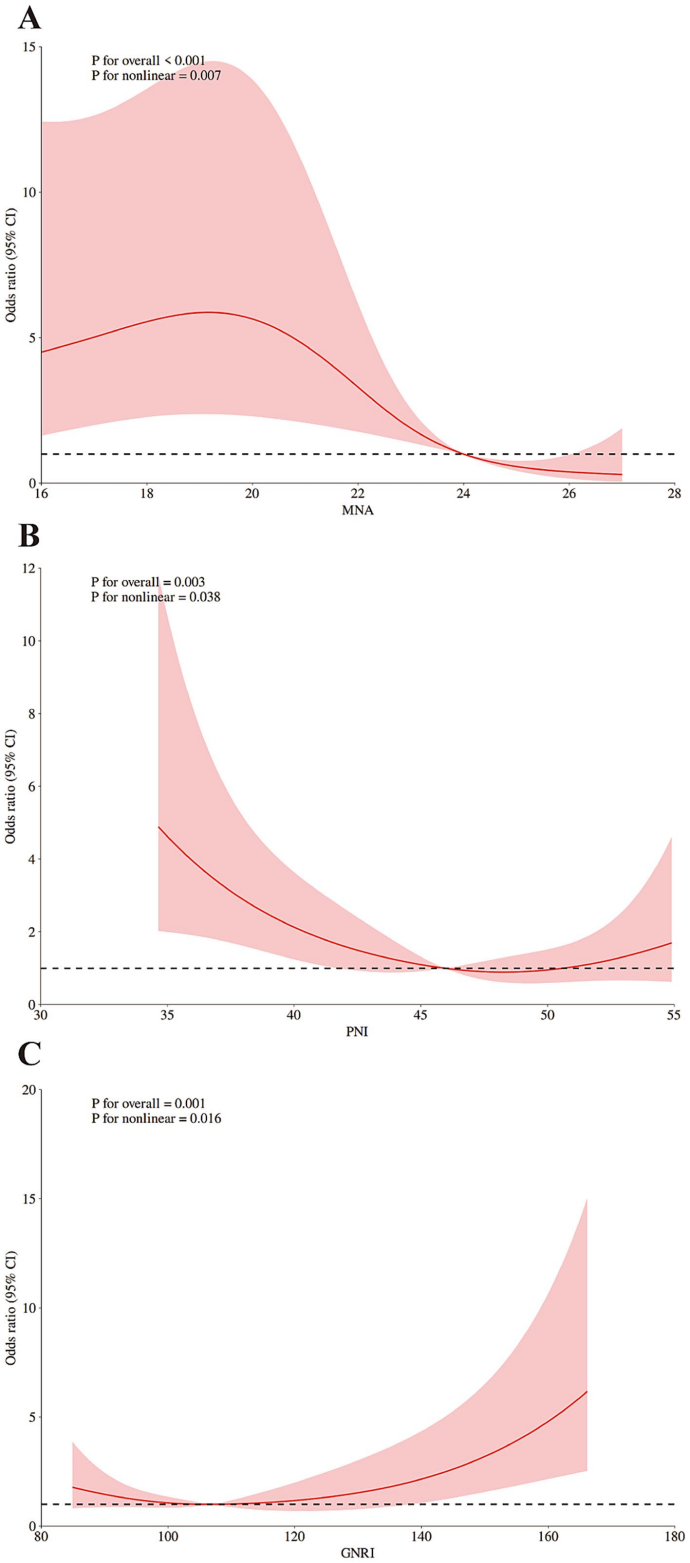
Restricted cubic spline (RCS) analysis of the dose–response associations: **(A)** between MNA and the risk of POD; **(B)** between PNI and the risk of POD; **(C)** between GNRI and the risk of POD. The reference point is the median of nutritional assessments scores; shading represents the 95%CI; the 5th, 50th, and 95th percentile of nutritional assessments scores were selected as knots, respectively. Adjusted for BMI (continuous), education years (continuous). MNA, Mini Nutritional Assessment; PNI, Prognostic Nutritional Index; GNRI, Geriatric Nutritional Risk Index; CRP, C-reactive protein.

Five-Factor Model (“Age + CRP + MNA + PNI + GNRI”): The addition of PNI and GNRI to the “Age + CRP + MNA” model did not significantly improve the AUC (0.807 vs. 0.810, *p* = 0.751 by DeLong’s test) (Table 4, Figure 3).

**Table 4:** Analysis of the efficacy of nutrition-related indicators and combinations of nutritional indicators in predicting the risk of postoperative delirium in the elderly after non-cardiac surgery.

Variables	Sensitivity (%)	Specificity (%)	AUC	95% CI	Cut off	*p* value
MNA	41	17	0.741	(0.67–0.81)	23.25	0.000
Age-MNA	74	72	0.760	(0.69–0.83)	0.168	0.000
CRP-MNA	70	81	0.807	(0.75–0.87)	0.125	0.000
Age-CRP-MNA	71	80	0.810	(0.75–0.87)	0.133	0.000
Age-CRP-MNA-PNI-GNRI	82	69	0.807	(0.74–0.87)	0.209	0.000

MNA, Mini Nutritional Assessment; PNI, Prognostic Nutritional Index; GNRI, Geriatric Nutritional Risk Index; CRP, C-reactive protein. CRP: C-reactive protein.

### Subgroup analysis

**Figure 4:**
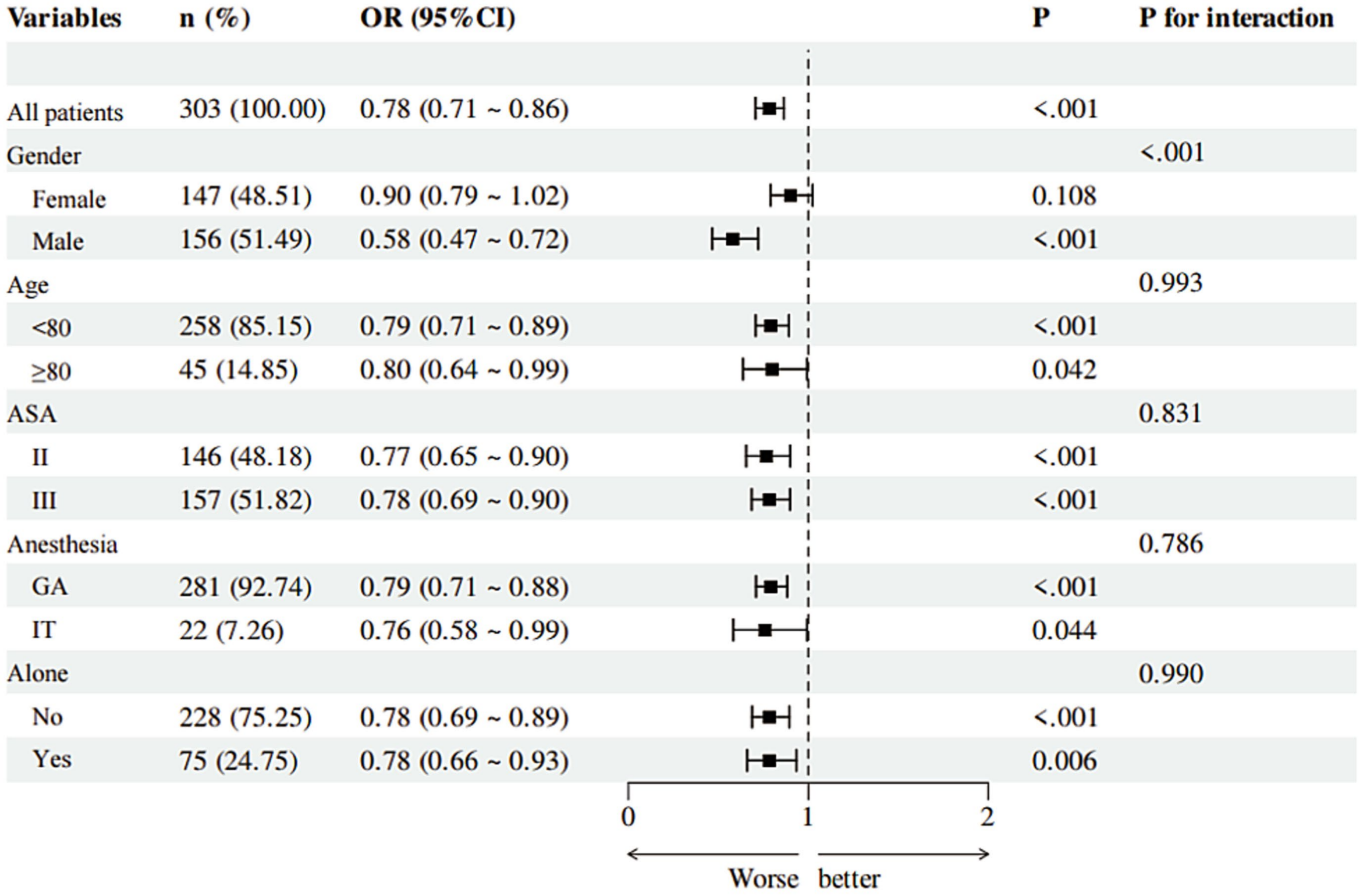
The results of subgroup analyses. OR, Odds Ratio; CI, Confidence Interval.

The protective effect of higher MNA scores (indicating lower POD risk) was consistent across most subgroups (age <80/≥80, ASA II/III, anesthesia type, living status). However, a significant interaction by sex was found (*p* < 0.001), with the association being strong and significant in males (OR = 0.58, 95% CI: 0.47–0.72, p < 0.001) but non-significant in females (OR = 0.90, 95% CI: 0.79–1.02, *p* = 0.108) (Figure 4).

## Discussion

This study is the first to systematically compare MNA, PNI, and GNRI in predicting POD among elderly patients undergoing non-cardiac surgery. Key findings reveal that MNA is the most effective single predictor of POD (AUC = 0.741), showing significantly better discriminative ability than the other two indices. Zhao et al. (22) found that the Mini Nutritional Assessment-Short Form (MNA-SF) was superior to the GNRI in predicting POD in elderly patients undergoing non-cardiac surgery. Liu et al. (24) also confirmed the correlation between the PNI and POD, which is consistent with our research results. In addition, advanced age, elevated CRP levels, and a lower MNA score independently predicted POD. These findings are consistent with previous studies that have established age, inflammatory status, and nutritional status as significant risk factors for POD (29, 30). Critically, the predictive model including age, CRP, and MNA achieved the highest accuracy (AUC = 0.810). Notably, adding either PNI or GNRI to this model did not significantly improve its performance. Finally, we observed a sex-specific modifying effect: the protective association between a higher MNA score and reduced POD risk was significantly stronger in male patients than in females.

The superior predictive performance of the MNA over the PNI and GNRI can be attributed to fundamental differences in their design and the domains they assess. The PNI and GNRI are predominantly biochemical and anthropometric indices, relying on serum albumin and lymphocyte count or body weight. While useful, these parameters can be confounded in the perioperative period by factors such as fluid shifts, systemic inflammation (which causes pseudo-hypoalbuminemia), and conditions like edema or sarcopenia that distort weight-based measures (31, 32). In contrast, the MNA provides a holistic assessment by incorporating critical functional and psychosocial dimensions—including mobility, neuropsychological status, depression, and dietary habits (33)—which are themselves well-established independent risk factors for POD (34–36). Therefore, the MNA likely captures a broader spectrum of vulnerability, effectively identifying patients who are not only biochemically malnourished but also functionally and cognitively frail. This multifaceted nature of the MNA makes it a more robust tool for predicting a multifactorial syndrome like postoperative delirium.

A significant sex interaction was observed: MNA conferred strong protection in males but not in females. This finding may reflect a higher inherent neuropsychiatric vulnerability in elderly males undergoing surgery, which could be further exacerbated by malnutrition. In the study by Kokras et al. (37), it was observed that these sex differences may originate from inherent neurobiological divergences. Estrogen confers notable neuroprotective effects in females, such as by modulating monoaminergic neurotransmitters (e.g., serotonin and dopamine) and amino acid levels in the prefrontal cortex and hippocampus, thereby enhancing neural resilience and mitigating the adverse effects of stress. In contrast, males exhibit greater neuroendocrine vulnerability, demonstrated by significantly exacerbated depression-like behaviors and more pronounced deterioration in neurochemical markers—such as hippocampal serotonin levels—following the loss of gonadal hormones. These findings suggest that elderly males may be more susceptible to impairment of the neuropsychiatric system when confronted with challenges such as surgical stress and malnutrition, potentially leading to a markedly increased risk of delirium (38). Conversely, neuroprotective effects of estrogen in females (37, 39) as well as sociocultural factors that influence nutritional status and health-seeking behaviors, might buffer against the impact of suboptimal nutrition, explaining the weaker association in this group. Future research should explore the interplay between sex hormones, nutrition, and delirium risk to inform sex-stratified preventive strategies.

CRP’s predictive value (OR = 1.06) highlights neuroinflammation’s key role in POD pathogenesis (40, 41). Advanced age (OR = 1.07) is a well-established risk factor because it causes neuroimmune dysregulation and blood–brain barrier compromise, which increase brain susceptibility to inflammatory insults (42–46). The “Age + CRP + MNA” model integrates three key biological domains: physiological reserve (represented by Age), inflammatory burden (CRP), and nutritional/functional status (MNA). Its high accuracy (AUC = 0.810) and reliance on readily available clinical bedside data (avoiding complex lab tests like PNI/GNRI) make it clinically practical.

The identified MNA threshold (≤24) provides a clinically actionable value for preoperative risk stratification. This model facilitates an efficient hierarchical screening strategy. Initial screening uses the MNA preoperatively, with scores ≤24 indicating elevated risk. Subsequently, for MNA-positive patients, refined risk assessment incorporates CRP measurement and considers patient age. Finally, targeted interventions can be directed toward high-risk patients. These interventions include preoperative anti-inflammatory nutritional support, such as *ω*-3 fatty acid-enriched diets (47–49). High-risk patients are defined by criteria including age ≥76 years, MNA score ≤22, and CRP ≥ 3.82 mg/L.

Several important limitations should be noted. First, because the study was conducted at a single center in Shanxi province with a predominantly Han Chinese cohort, the generalizability of the findings may be limited. Second, although all assessors in this study received standardized training to minimize assessment discrepancies, this potential bias cannot be fully eliminated. Third, the potential influence of unmeasured confounders [e.g., anesthesia depth (50), postoperative analgesia/opioid use (51)] cannot be excluded. Fourth, our inflammatory profiling was limited to CRP, which may not fully capture the complexity of the neuroinflammatory response. Fifth, our study focused on a comparison of three nutritional assessment tools (MNA, PNI, GNRI) and did not include other internationally common nutritional screening tools, such as the Nutritional Risk Screening 2002 (NRS-2002) or the Malnutrition Universal Screening Tool (MUST).

Future research should prioritize multicenter validation of the predictive model. And future studies could employ more objective methods to supplement the MNA, such as utilizing food diaries, involving family members in dietary recall, or developing novel assessment tools that integrate more objective biomarkers to complement the subjective elements. Mechanistic investigations are essential to elucidate the “nutrition-neuroinflammation” axis underlying POD. These should incorporate neuroinflammatory markers such as IL-6, S100β and TNFα (41). Future study directly comparing the predictive value of both screening and assessment tools would provide a more comprehensive clinical picture and further validate our findings. Crucially, Prospective interventional trials are also warranted to evaluate personalized nutritional strategies, such as high-protein and anti-inflammatory diets, based on MNA stratification. Furthermore, it is critical to elucidate the biological and socioracial mechanisms behind the observed sex disparity in MNA’s predictive efficacy. Risk assessment and intervention strategies should consider sex differences, as nutritional optimization may provide greater preventive benefits for male patients due to the observed effect modification.

## Conclusion

MNA is the best tool to assess nutrition and predict POD in elderly patients undergoing non-cardiac surgery. The combination of age, CRP, and MNA score forms a highly accurate (AUC = 0.810) and clinically practical predictive model. This model facilitates preoperative identification of high-risk patients, enables targeted preventive strategies such as nutritional optimization, and supports efficient resource allocation in perioperative care for the growing elderly surgical population.

## Data Availability

The raw data supporting the conclusions of this article will be made available by the authors, without undue reservation.
