# Supplementary material for: The predictive value comparison of the different nutritional assessment tools for postoperative delirium in elderly patients after non-cardiac surgery
Source: Front Nutr. 2025 Oct 21;12:1673973. doi: 10.3389/fnut.2025.1673973 (PMC12584556; doi:10.3389/fnut.2025.1673973)
Supplement: Supplementary file 1 [file Table_1.pdf]

STROBE Statement—Checklist of items that should be included in reports of ***cohort studies***

	Item No	Recommendation	Page (the section headings)
**Title and abstract**	1	(*a*) Indicate the study's design with a commonly used term in the title or the abstract	Title
		(*b*) Provide in the abstract an informative and balanced summary of what was done and what was found	Abstract
**Introduction**			
Background/rationale	2	Explain the scientific background and rationale for the investigation being reported	Backgroud
Objectives	3	State specific objectives, including any prespecified hypotheses	Backgroud
**Methods**			
Study design	4	Present key elements of study design early in the paper	Study Design and Population
Setting	5	Describe the setting, locations, and relevant dates, including periods of recruitment, exposure, follow-up, and data collection	Study Design and Population
Participants	6	(*a*) Give the eligibility criteria, and the sources and methods of selection of participants. Describe methods of follow-up	Study Design and Population
		(*b*) For matched studies, give matching criteria and number of exposed and unexposed	Study Design and Population
Variables	7	Clearly define all outcomes, exposures, predictors, potential confounders, and effect modifiers. Give diagnostic criteria, if applicable	Data Collection
Data sources/ measurement	8*	For each variable of interest, give sources of data and details of methods of assessment (measurement). Describe comparability of assessment methods if there is more than one group	Nutritional Assessment
Bias	9	Describe any efforts to address potential sources of bias	Statistical Analysis and Sample size
Study size	10	Explain how the study size was arrived at	
Quantitative variables	11	Explain how quantitative variables were handled in the analyses. If applicable, describe which groupings were chosen and why	Statistical Analysis and Sample size
Statistical methods	12	(*a*) Describe all statistical methods, including those used to control for confounding	Statistical Analysis and Sample size
		(*b*) Describe any methods used to examine subgroups and interactions	
		(*c*) Explain how missing data were addressed	Statistical Analysis and Sample size
		(*d*) If applicable, explain how loss to follow-up was addressed	
		(*e*) Describe any sensitivity analyses	
**Results**			
Participants	13*	(a) Report numbers of individuals at each stage of study—eg numbers potentially eligible, examined for eligibility, confirmed eligible, included in the study, completing follow-up, and analysed	Characteristics of Participants
		(b) Give reasons for non-participation at each stage	Characteristics of Participants
		(c) Consider use of a flow diagram	
Descriptive data	14*	(a) Give characteristics of study participants (eg demographic, clinical, social) and information on exposures and potential confounders	Characteristics of Participants
		(b) Indicate number of participants with missing data for each variable of interest	Characteristics of Participants
		(c) Summarise follow-up time (eg, average and total amount)	Characteristics of Participants
Outcome data	15*	Report numbers of outcome events or summary measures over time	
Main results	16	(*a*) Give unadjusted estimates and, if applicable, confounder-adjusted estimates and their precision (eg, 95% confidence interval). Make clear	Univariate and Multivariate Analysis of POD Predictors

		which confounders were adjusted for and why they were included<br>(*b*) Report category boundaries when continuous variables were categorized<br>(*c*) If relevant, consider translating estimates of relative risk into absolute risk for a meaningful time period	Predictive Performance of Nutritional Tools and Models
Other analyses	17	Report other analyses done—eg analyses of subgroups and interactions, and sensitivity analyses	Subgroup Analysis
**Discussion**			
Key results	18	Summarise key results with reference to study objectives	Discussion
Limitations	19	Discuss limitations of the study, taking into account sources of potential bias or imprecision. Discuss both direction and magnitude of any potential bias	Discussion
Interpretation	20	Give a cautious overall interpretation of results considering objectives, limitations, multiplicity of analyses, results from similar studies, and other relevant evidence	Discussion
Generalisability	21	Discuss the generalisability (external validity) of the study results	Discussion
**Other information**			
Funding	22	Give the source of funding and the role of the funders for the present study and, if applicable, for the original study on which the present article is based	Funding

*Give information separately for exposed and unexposed groups.

Note: An Explanation and Elaboration article discusses each checklist item and gives methodological background and published examples of transparent reporting. The STROBE checklist is best used in conjunction with this article (freely available on the Web sites of PLoS Medicine at http://www.plosmedicine.org/, Annals of Internal Medicine at http://www.annals.org/, and Epidemiology at http://www.epidem.com/). Information on the STROBE Initiative is available at http://www.strobe-statement.org.
